# The Role of Measurable Residual Disease (MRD) in Hematopoietic Stem Cell Transplantation for Hematological Malignancies Focusing on Acute Leukemia

**DOI:** 10.3390/ijms20215362

**Published:** 2019-10-28

**Authors:** Anna Czyz, Arnon Nagler

**Affiliations:** 1 Department of Hematology and Bone Marrow Transplantation, Wroclaw Medical University, Ludwik Pasteur 4, 50-367 Wroclaw, Poland; 2 Hematology Division, Chaim Sheba Medical Center, Tel Hashomer, Derech Sheba 2, 52-621 Ramat Gan, Israel

**Keywords:** measurable residual disease, acute myeloid leukemia, acute lymphoblastic leukemia, hematopoietic stem cell transplantation, multiparameter flow cytometer, polymerase chain reaction

## Abstract

The significance of measurable residual disease (MRD) in hematopoietic stem cell transplantation (HSCT) is well recognized in different hematological malignancies, but the evidence indicate that pre-transplant MRD status is of particular importance in acute lymphoblastic leukemia (ALL) and acute myeloid leukemia (AML). In ALL, inadequate response at the level of MRD is a commonly accepted risk factor for relapse and thus an indication for allogeneic HSCT. Similarly, growing evidence from the literature strongly suggest that MRD detected by multiparameter flow cytometry or molecular techniques should be also used for risk stratification in AML at the time of HSCT. Despite the well-defined association of MRD and outcomes of HSCT in acute leukemias, there are still many open issues such as the role of additional pre-transplant consolidation for MRD eradication, the ability of HSCT to overcome negative influence of MRD positivity on survival, the impact of conditioning regimen intensity on MRD clearance post HSCT, and transplantation outcomes or the selection of optimal donor with regards to MRD status. In addition, the role of MRD assessment in guiding post-transplant maintenance treatment should also be addressed in prospective trials. These open issues mostly awaiting further clinical studies will be discussed in our current review.

## 1. Introduction

In acute leukemias, measurable residual disease (MRD) is defined as the presence of residual leukemic cells in bone marrow (BM) or peripheral blood (PB) in patients who achieved morphologic complete remission (mCR). Notably, the new definition of LeukemiaNet for CR includes MRD negativity [1] indicating the crucial role and prognostic implication of the MRD status in modern leukemia therapy. Residual leukemic cells may be detected by multiparameter flow cytometry (MFC) or molecular techniques, including real-time quantitative polymerase chain reaction (RQ-PCR), digital droplet PCR, and next-generation sequencing (NGS), with different sensitivity and specificity for each of them.

The significance of MRD in hematopoietic stem cell transplantation is well recognized in different hematological malignancies, but the evidence indicate that pre-transplant MRD status is of particular importance in acute lymphoblastic leukemia (ALL) and acute myeloid leukemia (AML) as it correlates with the risk of relapse after hematopoietic stem cell transplantation (HSCT). The significance of MRD in acute leukemias has recently been highlighted in recommendations for diagnosis and management in ALL and AML published by different international groups of experts [1,2,3,4].

In ALL, inadequate response at the level of MRD is a commonly accepted risk factor for relapse and thus an indication for allo-hematopoietic stem cell transplantation (allo-HSCT) as was recently emphasized in the consensus paper by panel of international experts on behalf of the European Working Group for Adult Acute Lymphoblastic Leukemia (EWALL) and the Acute Leukemia Working Party of the European Society for Blood and Marrow Transplantation [2].

Similarly, growing evidence from the literature strongly suggests that MRD detected by MFC or molecular techniques should be also used for risk stratification in AML at the time of HSCT. The clinical significance of MRD status in AML has been confirmed recently by a position paper by the European LeukemiaNet (ELN) [1]. Indeed, according to 2017 ELN guidelines for diagnosis and management of AML, morphological CR was separated into two categories, depending on the MRD status, i.e., CR-MRD(−) and CR-MRD(+) with CR-MRD(−) being the ultimate goal in leukemic therapy.

Despite the well-defined association of MRD and outcomes of HSCT in acute leukemias, there are still many open issues such as the role of additional pre-transplant consolidation for MRD eradication, the ability of HSCT to overcome positive MRD by achieving MRD negativity and the potential price in term of toxicity and transplant related mortality, the impact of conditioning regimen intensity on MRD clearance post HSCT, and transplantation outcomes or the selection of optimal donor with regards to MRD status. In addition, the role of MRD assessment in guiding post-transplant prophylactic and pre-emptive treatment should also be addressed in prospective trials. These open issues mostly awaiting further clinical studies will be discussed in our current review.

## 2. The Role of MRD in HSCT for ALL

In acute lymphoblastic leukemia, detection of residual disease persisting below the mCR threshold of 10^2^ is the most important adverse prognostic factor for treatment outcome, independent from “the old” classical risk factors identified at diagnosis [5,6,7,8]. MRD detection by flow cytometry or molecular techniques in the post-induction phase of treatment in Ph-negative ALL is the only widely recognized and accepted risk factor considered an indication for HSCT by most of the research groups [5,6,7,8,9], as was just very recently highlighted in the consensus paper of EWALL and AWLP EBMT experts [2]. In daily clinical practice, MRD is measured with EuroFlow-based 8-color flow cytometry with the use of antibody panels and analysis tools that enable discrimination between residual leukemic cells and normal B- and T-cell precursors in bone marrow [10]. PCR and next-generation sequencing (NGS) are used to detect and monitor the fusion genes’ transcripts present in 30–40% of B- and T-ALL or to track clonal rearrangements of immunoglobulin or T-cell receptor genes, what is applicable in up to 90% of patients with B- and T-ALL. Bone marrow is preferred over peripheral blood for MRD measurement, since the blood MRD level are significantly lower compared to bone marrow, especially in B-ALL [11,12,13].

Sensitivities of clone-specific detection of immunoglobulin (Ig) and T-cell receptor (TCR) gene rearrangements using polymerase chain reaction (Ig/TCR PCR) range from 10^−4^ to 10^−5^ [14]. However, the precise identification of junctional region sequences that are patient-specific targets for MRD detection is required in each ALL in order to design clone-specific oligonucleotides, which is only possible in experienced molecular hematology laboratories. The major advantages of RQ-PCR analysis of the Ig/TCR gene rearrangements are its broad applicability, the usage of DNA as a stable analytical sample and high degree of PCR primers and protocols standardization [15,16,17]. As the result of standardization of Ig/TCR PCR analysis for MRD detection, the guidelines for the analysis and interpretation of RQ-PCR data was established by the EuroMRD Consortium more than a decade ago [15]. Yet, an important disadvantage of this method is the frequent occurrence of clonal evolution between diagnosis and relapse, which may result in false-negative PCR results [18]; therefore, it is recommended to use at least two IG/TCR gene targets for MRD monitoring [14,18]. The other potential obstacles are the subclones that may exist at diagnosis and lead to incorrect estimation of MRD level. Taking into consideration these pitfalls of PCR, NGS appears an important new tool for MRD monitoring that may allow overcoming RQ-PCR limitations [19]. With the use of universal primers, NGS enables more comprehensive detection of any Ig/TCR rearrangement at the same time [20,21]. Additionally, depending on the amount of DNA, NGS reaches sensitivity of 10^−5^ to 10^−6^ [22]. Some authors have reported that NGS is more specific than RQ-PCR in the prediction of ALL relapse [19,21,23]. However, in contrast to RQ-PCR, the guidelines for the analysis and interpretation of NGS data have not been established so far. Very recently, the EuroClonality-NGS working group reported the results of multicentre standardization and validation of NGS-based Ig/TCR markers’ identification in lymphoid malignancies for subsequent MRD analysis in ALL [20]. The five reference laboratories compared the results of Ig/TCR NGS analyses with the results of PCR and Sanger-sequencing for clonal products. NGS primer sets identified on average 4% more clonal sequences per patient than Sanger-sequencing. In addition, NGS enabled correct sequencing of bi-allelic rearrangements and lower copy fusions in the presence of a background of polyclonal rearrangements. The authors conclude that EuroClonality-NGS developed Ig/TCR marker identification protocol, which allows for quality-controlled and comprehensive detection of clonal Ig/TCR rearrangements in ALL [20]. The summary of sensitivity and specificity of different molecular methods for MRD detection in ALL was presented in Table 1.

MRD positivity in post-induction therapy is the universal strongest risk factor for relapse, regardless of the heterogeneity in MRD definitions applied, techniques used, and the time point being assessed in the different studies. Its negative impact on leukemia-free survival (LFS) may be at least partially overcome by HSCT, as strongly suggested by the results of all three cooperative trials conducted by the GMALL, PETHEMA, and GRALL research groups [6,7,9]. In a pooled analysis of the GMALL 06/99 and 07/03 trials focused on prospective MRD monitoring by RQ-PCR directed to *TCR* and *Ig* gene rearrangements, it was demonstrated that the probability of disease-free survival (DFS) after 5 years was significantly higher for patients with persistent MRD > 10^4^ who underwent HSCT in first CR than for those patients that did not undergo HSCT in first CR (50% versus 16%, *p* = 0.004) [7]. Similar results were reported by the French/Belgium/Swiss group [9]. Furthermore, the reassessment of the GRAALL-2003 and GRAALL-2005 trials’ data showed that HSCT was associated with a longer relapse-free survival (RFS) in patients with postinduction MRD ≥ 10^−3^ (hazard ratio, 0.40) assessed by RQ-PCR. In contrast, no benefit of HSCT on RFS was demonstrated in good MRD responders [9].

Although outcomes of patients with persistent MRD who undergo HSCT is better compared with those who are treated with chemotherapy, the relapse rate after HSCT is significantly higher in MRD positive patients in comparison to those with undetectable MRD before transplant. Consequently, one can assume that eradication of MRD before HSCT may significantly improve the outcomes of transplant. A proof of principle is ALL. The efficacy of blinatumomab, a bispecific T cell–engager antibody in MRD eradication was evaluated in a single-arm study in adult patients with ALL in CR who exhibited MRD positivity after chemotherapy [26]. A complete MRD response was achieved by 78% of patients treated with blinatumomab. Over 60% of patients underwent HSCT in continuous CR. Among all patients, RFS was 54% at 18 months, with similar estimates with and without censoring for post-blinatumomab HSCT and chemotherapy. The authors concluded that these results compare favorably with published data for MRD-positive ALL. However, since a significant number of patients with a complete MRD response remained in long-term remission without subsequent HSCT, authors emphasized that the role of HSCT in this clinical setting should be determined in additional prospective studies [26].

The other issue is the role of pre- and post-transplant MRD monitoring in guiding maintenance therapy after HSCT. This approach is intensively investigated in Ph-positive ALL. The use of tyrosine kinase inhibitors (TKIs) in post-transplant maintenance treatment results in reduced relapse incidence and improved long-term outcomes of HSCT, as was demonstrated by several prospective and retrospective studies [27,28,29,30,31]. Nevertheless, the approach in patients with Ph-positive ALL after HSCT relies on the results of post-transplant BCR-ABl1 transcript assessment, as nicely summarized in the position statement from the Acute Leukemia Working Party of the European Society for Blood and Marrow Transplantation [3]. Patients with MRD(+) after HSCT should start TKI treatment as soon as possible, while patients with MRD(-)may be treated prophylactically or alternatively may be strictly monitored and receive TKI only after the detection of MRD in subsequent tests. In contrast to Ph-positive ALL, there are no data so far on post-transplant MRD eradication with novel targeted therapies in Ph-negative ALL.

### MRD Allows Revisiting Autologous Transplantation in ALL

Furthermore, the development of highly sensitive techniques for MRD assessment may allow for revisiting the role of autologous transplantation in ALL. On behalf of the Acute Leukemia Working Party of EBMT, Giebel et al. retrospectively compared autologous versus allogeneic transplantation with myeloablative conditioning in patients with Ph+ ALL in first molecular remission and found no differences in outcomes [32]. The authors concluded that, in the TKI era, autologous transplantation appears to be an attractive treatment option for patients with Ph-positive ALL potentially circumventing the short- and long-term consequences of allogeneic transplantation. The same investigators on behalf of the European Study Group for Adult ALL performed retrospective analysis on the role of autologous transplantation in the treatment of high-risk adult ALL, including both Ph-positive and Ph-negative ALL [33]. In a cohort of Ph-negative ALL, the estimated 5-year LFS was 57% for patients with MRD negative status (defined as MRD level < 0.1%) being 2-fold higher than the LFS probability for patients with MRD positive status at transplant. In multivariate analysis, high MRD level remained the only independent prognostic factor associated with an increased risk of failure. The authors concluded that the role of autologous transplantation in ALL need to be re-evaluated in further prospective trials.

## 3. The Role of MRD in HSCT for AML

In acute myeloid leukemia, genetic risk defined according to European LeukemiaNet (ELN) criteria is the most relevant factor in prognostication and decision-making processes [1,34]. However, in view of the growing evidence on the role of MRD status for AML treatment outcomes, the experts of the ELN MRD Working Party stated in recently published document that measurable residual disease is an independent, post-remission prognostic factor in AML that is important for risk stratification and treatment decision-making process [4].

The genetic and immunophenotypic diversity of AML is a major challenge for introducing an MRD assessment into daily clinical practice. In view of these difficulties, the ELN MRD Working Party has published guidelines for the use of MRD in clinical practice [4]. The two methods that are currently most widely applied for MRD assessment are MFC and RQ-PCR. However, the new technologies of digital PCR and NGS emerge as particularly useful in AML [35,36,37]. Each of them differs in sensitivity, specificity, and the applicable patient populations, what has been nicely summarized in several review articles by the experts in the field [36,37].

The most widely employed technique, applicable for more than 90% of the AML patients, remains the MFC with a sensitivity between 10^−3^ and 10^−5^ [36,38,39]. The 10^−5^ sensitivity can be reached mostly in experienced flow cytometry laboratories. MRD monitoring with MFC relies on defining leukemia-associated immunophenotypes (LAIPs) at diagnosis which is required in order to be able to detect MRD in the post-remission phase of the treatment. Alternatively, MRD assessment may be based on an analysis that screens for established aberrant differentiation/maturation immunophenotypic profiles at follow-up that is termed “different-from-normal” (DfN) approach [40]. The third, yet developmental method for MRD assessment focuses on the detection of leukemic stem cell (LSC) in the compartment of the CD34+CD38− stem/progenitor cells [36,41,42,43] The group from the Netherlands had nicely defined aberrant marker expression patterns on the AML CD34+CD38− stem cell compartment allowing distinguishing between the malignant and the normal stem cell compartment both at diagnosis as well as in remission [43]. Moreover, focusing on LSC frequency in adults with AML, Terwijn M. et al. demonstrated that higher percentages of neoplastic CD34+CD38− cells at diagnosis as well as in CR post various courses of therapy strongly correlates with shorter patient survival, while the neoplastic parts of the CD34+CD38+ and CD34− putative stem cell compartments had no prognostic impact [42].

The main technical limitations of MRD monitoring by MFC result from (1) the hemodilution of the BM samples; (2) difficulties in proper gating of both pure WBC and blast cells, and (3) biological heterogeneity of the leukemic population. The 2017 ELN guidelines recommend RQ-PCR or NGS as alternative methods for MRD assessment. PCR-based methods (e.g., RQ-PCR and digital droplet PCR) depend on identification of pretreatment-AML-associated mutation at diagnosis [4]. RQ-PCR, to some extent, the clinical “gold-standard assay”, is characterized by high interlaboratory reproducibility, high specificity and sensitivity for leukemic cells, as well as decreased contamination risk [37,40,44]. However, it can be applied only to patients expressing validated molecular targets that are stable while on therapy. These targets include *NPM1* mutation, *RUNX1-RUNX1T1* and *CBF-MYH11* in CBF-AML, and *PML-RARA* in acute promyelocytic leukemia [4,36,45], for which real-time quantitative reverse transcriptase polymerase chain reaction (RT-qPCR) methods have been standardized by the Europe Against Cancer (EAC) consortium [46] with a sensitivity threshold of 10^−4^ to 10^−6^. However, these mutations accounts for no more than 50% of AML patients; in other words, RT-qPCR based MRD monitoring is not applicable for at least half of the AML patients [34,37]. The other mutations found in AML, such as *FLT3*-ITD (ITD, internal tandem duplication), *FLT3*-TKD (TKD, tyrosine kinase domain), *NRAS*, *KRAS*, *IDH1*, *IDH2,* and *MLL*-PTD may be lost between diagnosis and relapse as a result of clonal evolution of leukaemia, thus being unstable throughout treatment. Therefore, ELN guidelines advise against using them as single markers, but they may be used to detect MRD in combination with other mutations [4]. In cases without identifiable molecular targets and with unavailable flow cytometric assay, ELN guidelines recommend the Wilms tumor gene (*WT1*) expression analysis in MRD assessment with the use of standardized and validated *WT1* assay developed by ELN researches, who established reference ranges for *WT1* expression in normal blood and bone marrow [47]. *WT1* encodes a transcription factor overexpressed in approximately 90% of AML patients, but the assessment of its expression for MRD monitoring has remained controversial due to limited data from large, controlled studies, as well as undetermined precise cut-off values and time-points for analysis. However, very recently researches from South Korea published a study on the optimal time-points and thresholds for the bone marrow *WT1* expression assessment by real-time PCR with the use of the ELN normalized method in a large cohort of AML patients undergoing allo-HSCT [48]. The authors have found *WT1* MRD assay useful for identifying patients with high risk of AML relapse.

Digital droplet PCR (ddPCR) is the new high-throughput technology that enables an absolute quantification of clonally amplified target genes without a reference standard amplification curve that is needed in conventional RQ-PCR. The ddPCR technique is characterized by high sensitivity exceeding 10^−3^, down to 10^−5^. It is applicable for monitoring a variety of *NPM1* mutations [49], as well as *DNMT3A* and *IDH* mutations [50] in AML at the time of CR with the possibility to monitor several mutations simultaneously. The major disadvantage highlighted by the experts is that very often some new assays have to be designed for each patient, since a single assay needs to be developed for specific base changes in the same gene [44,51].

NGS offers several advantages over PCR-based methods, such as the ability to study the entire leukemic genome, which can potentially be used in almost all AML patients. This molecular tool requires highly specialized bioinformatic approaches in order to identify all emerging clinically relevant variants or alleles. NGS used to detect MRD measures the variant allele frequency (VAF) of mutations. The sequencing approaches include Whole Genome Sequencing (WGS), Whole-Exome Sequencing (WES) and targeted-gene sequencing technology [44]. The sensitivity of standard NGS platforms is limited to approximately 1–5% VAF. This limitation of sensitivity may be overcome in targeted-gene sequencing NGS by so called “deep sequencing” of selected alleles (such as *NPM1* gene) and after applying error-correcting statistical models for raw sequencing data acquisition [52]. Jongen-Lavrenic et al. have shown recently that at least one mutation could be detected at diagnosis in almost 90% of AML patients with the use of targeted NGS, and the persistence of most of these mutations during CR was proved to be associated with an increased risk of relapse [53]. However, the interpretation of NGS-MRD evaluation requires consideration of clones associated with age-related clonal haematopoiesis, also known as clonal haematopoiesis of undetermined potential (CHIP). CHIP is observed in approximately 10% of normal individuals older than 65–70 years of age, as was clearly demonstrated by two research groups which analyzed data from whole-exome sequencing of DNA of more than 10,000 individuals, unselected for cancer or hematologic phenoptypes [54,55]. Mutations of epigenetically active genes such as *DNMT3A*, *TET2*, and *ASXL1* (abbreviated as DTA) are known to be most frequent in CHIP, and thus may occur in healthy individuals with an increased incidence with age [54,56]. Consequently, their persistence after treatment was not found as correlated with an increased relapse rate in AML and did not have a prognostic role [53,57]. The summary of sensitivity and specificity of different molecular methods for MRD detection in AML is presented in Table 2.

### 3.1. MRD Assessment as a New Tool in AML for Post-Remission and Pre-Transplant Risk Stratification

MRD assessment in the post-induction phase of the treatment of an AML patient is emerging as a new tool that can be used to predict poor outcome in standard-risk AML patients who may benefit from HSCT in the first CR. In this regard, the UK National Cancer Research Institute (UK NCRI) AML Working Group has evaluated prospectively the predictive value of MFC-MRD after first and second course of intensive chemotherapy in patients with AM [58]. The patients who achieved CR after induction chemotherapy were subdivided into MRD (+) and MRD (−). MRD positivity after second course of chemotherapy appeared as a significant adverse prognostic factor for OS, being more discriminatory in good and standard risk patients, compared to the high-risk group. The prognosis associated with positive MRD was as dismal as the established poor prognosis in clinically defined partial remission or resistant disease. The UK NCRI AML Working Group also addressed the issue whether molecular profiling and sequential RT-qPCR-based monitoring of MRD in patients with *NPM1*-mutated AML treated with intensive chemotherapy may provide prognostic information useful in routine practice [45]. The results of the study demonstrated that the presence of MRD as determined by detection of *NPM1*-mutated transcripts after the second chemotherapy cycle in peripheral blood (PB) of patients in CR was the only significant prognostic factor. Notably as for the question of the timing of MRD assessment and the issue of bone marrow (BM) versus PB, assessment of MRD after the second chemotherapy cycle was more discriminatory than RT-qPCR positivity in PB at any other time point or assessing it in bone marrow samples. Moreover, the prognostic significance of the MRD status in the peripheral blood after the second chemotherapy cycle was also confirmed in the validation cohort. In fact, the presence of MRD compared to the absence of *NPM1*-mutated transcripts was associated with an increased 2-year cumulative incidence of relapse (70% versus 31%, *p* = 0.001) and a lower OS rate (40% versus 87%, *p* = 0.001). Additionally, MRD positive status after second cycle of chemotherapy in a subgroup of patients not harboring the *FLT3-ITD* mutation was also predictive for a very poor outcome with 2-year CI of relapse of 76% compared to 33% for MRD negative patients. Since the number of MRD positive patients who underwent HSCT in the first CR was small, the authors were not able to confirm a significant benefit of transplantation for those patients. This important question is currently addressed in an ongoing randomized phase III trial being conducted by the UK NCRI AML Working Group. The above UK NCRI AML findings are in line with the results of the Acute Leukemia French Association study, in which *NPM1*-mutated transcripts were assessed in PB after induction chemotherapy [59]. Similar to the findings of UK NCRI AML Working Group, low reduction of *NMP1* (<4-log) after chemotherapy was associated with poor outcome. Importantly, the study results indicated that HSCT improved OS and DFS only in patients with low reduction of *NPM1*. This finding is further supported by the results published by Freeman et al. demonstrating that in the subgroup of standard-risk patients without *NPM1* mutations, only those with MRD+ benefit from HSCT in first CR [58].

Regardless of the beneficial effect of allogeneic transplantation improving the outcomes of MRD-positive patients with low genetic risk, the results of HSCT in patients with intermediate and high-risk AML and positive MRD, for whom HSCT is the standard treatment approach, seem to be dismal, much worser than in patients with CR-MRD(−). Indeed, there is growing evidence confirming that also in AML, pre-transplant MRD assessment by either MFC or molecular techniques can be used for risk stratification at the time of HSCT following either myeloablative or reduced intensity conditioning [60,61,62,63,64]. Toward this end, the data emerging from the Fred Hutchinson Cancer Research Center indicate that pre-transplant MRD status assessed by MFC correlate with DFS, relapse rate (RI), and even overall mortality post-allogeneic transplantation with myeloablative conditioning (MAC) [60]. The authors also assessed the significance of MRD in AML patients undergoing MAC HSCT in first and second CR [65]. The negative impact of pre-transplant positive MRD on transplantation outcome was similar for patients in first or second remission (CR1 and CR2), while MRD negative status was associated with excellent transplant outcomes in both CR1 and CR2. Furthermore, the recently published large retrospective study by the EBMT registry in AML patients undergoing HSCT, confirmed that MRD status measured as per transplanting centre policy correlates with relapse risk as well as with LFS and OS, regardless the intensity of conditioning regimen [62]. Of note, the authors also addressed the question of the possible diverse effect of the MRD status in different regimen intensity, i.e., MAC- versus RIC-based HSCT in older and younger patients. The results of the study’s multivariate analysis indicated that the outcomes of MAC HSCT were better compared to RIC and non-myeloblative (NMA) HSCT only in MRD positive patients aged < 50 years. Overall, the results of Gilleece and colleagues suggested that in MRD negative patients, reduced intensity conditioning including RIC-/NMA-based transplantation may be the preferred choice in order to avoid MAC associated toxic sequelae while providing sufficient graft-versus-leukemia (GVL) effect. These findings await confirmation in prospective studies.

The molecular profiling and MRD assessment introduced into the prognostic stratification of patients with AML is a beginning of a new era of MRD-directed therapy, which will most probably allow patient-tailored and precision-type medicine avoiding under- or over-treatment of AML patients in the near future. In this regard, an important and critique question is the role of post-remission consolidation therapy in patients in CR with positive and negative MRD.

### 3.2. Does Pre-Transplant Consolidation Chemotherapy Improve the Outcomes of HSCT in AML?

The retrospective analyses of both the EBMT and CIBMTR registries strongly suggest no advantage of post-remission consolidation chemotherapy pre-transplantation with either MAC or RIC preparative regimens. Supporting the no role for post-remission consolidation chemotherapy are data published by Cahn et al. on behalf of the EBMT registry for AML patients undergoing HSCT from a matched related donors with MAC [66], and more recently by Yeshurun et al. for AML patients undergoing RIC-based HSCT from a matched related or unrelated donor [67]. The results of both analyses, including large group of AML patients, indicate that pre-transplant consolidation does not improve outcomes of AML patients undergoing HSCT in terms of cumulative incidence of relapse and LFS. CIBMTR registry data analyses published by Tallman et al. and Warlick et al. reached the same conclusions regarding the role of consolidation before transplantation with either MAC or RIC, respectively, in AML achieving CR after first induction therapy [68,69]. However, the main limitation of the large registries data is the lack of information regarding pre-transplant MRD status. Based on the available data regarding the predictive value of MRD, it is conceivable that post-remission consolidation in MRD positive patients could eradicate MRD and achieving MRD negative status might result in improved transplantation outcome.

Additional important question is whether the kinetic of MRD clearance matter. Buccisano et al. analyzed the kinetics of reduction of MRD measured by MFC in bone marrow of AML patients treated with intensive chemotherapy according to the EORTC/GIMEMEA protocols [70]. The authors concluded that post-consolidation MRD status affect outcomes in terms of OS, LFS, and RR independent of post-induction MRD response, since patients with late MRD negativity achieved only after consolidation had the same outcome as the patients who achieved early remission and MRD negativity after the first induction course. Even more important, stressing the clinical advantage of achieving MRD negativity already after the first induction course is the fact that among 57 patients who were MRD(+) after induction, only 10 patients achieved MRD negativity after consolidation. Sustained MRD negativity is most probably of importance as well and is being currently the subject of various clinical studies. It is therefore of vast interest that, of a total of 35 patients with MRD negativity after induction, nine become MRD positive down the line indicating the future need to develop therapeutic means to keep patient’s MRD negative status. In total, the results of Buccisano et al. suggest once more that consolidation chemotherapy unfortunately is not helpful as it does not effectively eradicate MRD in AML patients undergoing allogeneic transplantation. In conclusion, the above mentioned data suggest that the prospective studies are warranted in order to define the role of MRD in directing post-remission therapeutic decision before HSCT in AML patients as it remains somewhat unclear whether patients in complete remission but still with positive MRD should proceed directly to transplant or should be treated with consolidation chemotherapy.

In subsequent publication, Buccisano et al. addressed the question whether the patients in CR with positive MRD may benefit from allogeneic transplantation [71]. In their study, Buccisano and colleagues from University Tor Vergata in Rome focused on a group of 81 patients with their AML treated according to the EORTC/GIMEMA protocol who underwent autologous or allogeneic HSCT while in complete remission but with positive MRD at the time of transplantation. The outcomes of allogeneic HSCT in terms of DFS, OS, and RI were significantly better than the results of autologous transplantation, with a 5-year DFS of 60% versus 19% and a 5-year relapse incidence of 18% versus 72%; thus, allogeneic HSCT in contrast to autologous HSCT may partially overcome the negative impact of MRD and enable a cure for a substantial number of patients with post-chemotherapy MRD positivity.

Because MRD emerges as a strong and independent risk factor for AML recurrence after allogeneic HSCT, a question arises regarding the prevention of post-transplant leukemia relapse in patients transplanted in CR-MRD(+) or in those with re-appearance of MRD after transplant. The increasing knowledge of both the molecular landscape of AML on one hand and better understanding of the biology of the GvL effect on the other opens the new perspectives on post-transplant MRD-targeted therapies, what has been very recently elegantly presented in expert review from the ALWP of the EBMT [72]. Potentially effective therapies for prevention of AML relapse after HSCT are currently being investigated in ongoing clinical trials including hypomethylating agents, i.e., azacytidine and decitabine, histone deacetylase inhibitor, ponabinostat, and hedgehog inhibitors. Moreover, in AML with *FLT3* internal tandem duplication (*FLT3* ITD) inhibitors of FLT3, sorafenib, midostaurin, quizartinib, gilteritinib, and others, are also intensively assessed in active phase II–III trials [72].

### 3.3. Selection of the Optimal Donor with Regard to the MRD Status—Does it Matter?

In the context of suboptimal outcomes of HSCT in AML patients in complete remission but with positive MRD, the issues of whether selection of the type of graft or donor will improve results and whether the donor should differ in MRD positive as opposed to the MRD negative AML patient have been investigated. In other words, what is the optimal donor for MRD positive AML patients and whether alternative donors or mismatched donors with broader HLA disparities and thus theoretically stronger GVL are of advantage in eradicating MRD have been studied. Indeed, the impact of donor type in AML patients with pre-transplant MRD positivity was the subject of few retrospective studies also addressing the prognostic significance of MRD status for those type of transplantation. Specifically, the group from Seattle compared retrospectively the results of a first HSCT from an unrelated cord-blood (CB) donor versus transplantation from matched unrelated donor (MUD) or from an HLA-mismatched unrelated donor (mMUD) following myeloablative condition in a large group of patients with AML and myelodysplastic syndrome (MDS) who had MRD determined by MFC before transplantation [73]. Interestingly, the risk of death in patients with MRD positive status before transplantation was significantly higher when transplanted from mMUD compared to those when transplanted from CB donor. Moreover, the relapse risk was higher after HSCT from both mMUD and MUD compared to those from CB donor. In contrast, the differences in outcomes of HSCT from CB donor, mMUD, and MUD in MRD negative patients were not significant. Importantly, in the subgroup of MRD positive patients, the survival rate after transplantation from CB donor was higher than the survival after transplant from mMUD, while not superior than survival after transplant from MUD. Therefore, in patients from ethnic minority groups for whom it may be difficult to find HLA-matched donor, the use of CB donor can allow not just to proceed rapidly to HSCT but equally importantly to eradicate a positive MRD status.

### 3.4. MRD Allows Revisiting Autologous Transplantation in AML

The molecular profiling and MRD assessment introduced into the prognostic stratification of patients with AML seem to begin a new era of MRD-directed therapy, which may allow avoiding under- or over-treatment of AML patients in the near future.

The development of highly sensitive and specific MRD assessment methods seems to lead to the rejuvenation of the concept of autologous transplantation in AML. Autologous transplantation can potentially allow a selected group of patients, especially with low and potentially intermediate genetic risk, to avoid over-treatment with allogeneic transplantation, providing 50–60% probability of long-term LFS [74]. The recently published excellent outcomes of autologous transplantation in MRD negative patients with low or intermediate genetic risk [75,76,77,78] prompt re-evaluating the role of autologous transplantation in AML. In this regard, a group from China compared retrospectively autologous to allogeneic HSCT in AML patients with favorable or intermediate genetic risk [76]. The authors found that autologous and allogeneic HSCT offered comparable DFS to patients with negative MRD after one course of consolidation. In contrast, the outcomes of autologous HSCT in patients with positive MRD was inferior because of high risk of relapse [76]. Very recently, Venditti et al. published the results of a multi-center, prospective, MRD-oriented, risk-oriented, prospective clinical trial on behalf of the Fundpo Italiano Malattie Hattatologiche dell’Adulto (GIMEMA) Foundation at AML [79]. Patients were categorized as favorable, intermediate, or high risk based on cytogenetic and genetic abnormalities according to the National Comprehensive Cancer Network classification. After the completion of the consolidation phase, patients with favorable risk were allocated to autoHSCT while those with high risk to allo-HSCT. Patients with intermediate risk were directed either to allo-HSCT or autoHSCT depending on their MRD status. The two-year DFS was 61% in patients with favorable risk, and 45% in those with poor risk. In the intermediate risk group, the two-year DFS in patients with MRD negative and positive status was 61% and 67%, respectively. The authors conclude that allo-HSCT prolongs OS and DFS in patients with intermediate risk and MRD positive status to the figures observed in patients with favorable risk. Moreover, the authors also suggested that autoHSCT may be considered in patients with favorable risk, guaranteeing similar results to those achieved with multiple courses of cytarabine. Of note, in patients with intermediate risk and negative MRD it may be possible to skip allo-HSCT. Two other groups studied the significance of residual leukemic cells detected in cryopreserved autografts. In a retrospective analysis of AML patients who underwent autologous HSCT at University of California, MRD assessed by RQ-PCR for a multigene panel in cryopreserved autograft appeared to be highly predictive for relapse in patients with known chromosomal abnormalities or mutations, including *WT1*, mutated *NPM1*, t(8;21), inv(16), and t(15;17) [77]. In the first year after autologous HSCT, there were no relapses in MRD-negative patients compared to 83% relapse incidence in MRD-positive patients. However, the results of the study suggest that MRD detection by MFC in cryopreserved autografts have limited predictive value for AML relapse after autologous HSCT; similarly, assessment of MRD in autografts by only *WT1* analysis alone was with limited predictive value as well. Intriguingly, opposite results regarding the prognostic significance of testing the MRD status of the autograft by *WT1* as the only tumor mutation were published by the group from Milan [80]. They found that *WT1* expression level measured by RQ-PCR in autologous peripheral blood stem cells (PBSC) from leukapheresis discriminates the risk of relapse and is associated with DFS and OS after autologous HSCT.

In conclusion, there is growing evidence that results of autologous HSCT in patients with low and intermediate risk AML are very good, provided that MRD status is negative before transplant.

## 4. Conclusions

Measurable residual disease has been used to guide therapeutic decisions and therapies in pediatric acute lymphoblastic leukemia for more than two decades. The lessons learnt from pediatric ALL were gradually incorporated into adult ALL and AML therapeutic strategies. Data are currently emerging for both adult ALL and AML that, similar to pediatric ALL, MRD is a new, extremely important prognostic factor in both types of adult acute leukemia. MRD should be most probably incorporated in the treatment decision process for both adult ALL and AML, similar to being used in pediatric ALL, although there are still many open questions and some controversies discussed in our review. The widely available MFC and RQ-PCR methods for MRD measurement may allow the introduction of MRD into the risk stratification in AML in daily clinical practice in the nearest future. It may lead to treatment de-escalation in patients with intermediate genetic risk who achieve CR with MRD negativity during the post-remission therapy. The era of MRD-directed therapy in AML may even lead to revisiting and new perspectives for autologous transplantation for patients with low or even intermediate risk AML achieving MRD negative status, although this approach is controversial as GVL is of major importance in curing AML. On the other hand, there is strong evidence that patients with *NPM1* mutation who are MRD positive after the second course of chemotherapy should be considered for early allogeneic transplantation. As for the role of pre-transplant consolidation, there are still controversies about its necessity in patients that achieved remission but are MRD positive and are about to go allogeneic transplantation, since there is no evidence that consolidation can eradicate MRD in AML. On the other hand, there are strong data regarding the independent impact of pre-transplant MRD status on the outcomes of HSCT, with similar significance whether MAC and RIC were applied. The broad availability and predictive capabilities of MRD as summarized in the current review may open new perspectives for novel targeted therapies both for pre-transplant MRD eradication as well as for post-transplant maintenance therapy in both ALL and AML. Lastly, as for the question of optimal selection of donor depending on AML patients’ MRD status, the published data, although on limited number of patients, may suggest that the negative impact of MRD on transplant outcomes might be partially overcome by allotransplant from cord blood graft or haploidentical donors in comparison to transplantation from unrelated or sibling donors. However, due to small size of study group and retrospective nature of the analysis these somewhat provocative data should be confirmed in further studies.

## Figures and Tables

**Table 1:** A comparison of molecular methods for measurable residual disease detection in acute lymphoblastic leukemia.

Method	Molecular Target	Applicability	Sensitivity Threshold	Advantages	Limitations	Recommendations for MRD Detection	Standardization
RQ-PCR	Ig/TCR rearrangement	Up to 85–90%	10^−4^ to 10^−5^	-high sensitivity -broad applicability -high degree of standardization	-affected by clonal evolution -it is recommended to use at least two IG/TCR gene targets for MRD monitoring [14,18] -time-consuming	EWALL/ALWP EBMT [2] A panel of international experts [14]	EuroMRD Consortium (formerly ESG-MRD-ALL) [15] Euroclonality/Biomed-2 [17]
RQ-PCR	Fusion genes *(BCR1-ABL1, KMT2A/AF4)*	35–45%	10^−4^ to 10^−5^	-high sensitivity -easy to perform -not expensive -target stability throughout therapy	-applicable only to specific leukemia subtypes (e.g., ALL with *BCR-ABL1 or KMT2A-AF4* mutation)	EWALL/ALWP EBMT [2] A panel of international experts [14] (ALWP EBMT) [3]	Euro-MRD Consortium (RQ-PCR for the e1a2 *BCR-ABL1* transcript detection) [24] Burmaister et al. (DNA-based quantitative PCR for *MLL* rearrangement detection) [25]
NGS	Ig/TCR rearrangement	>95%	10^−4^ to 10^−6^	-high sensitivity -broad applicability -comprehensive detection of clonal Ig/TCR rearrangements (potential to identify clonal evolution)	-expensive-requirement of highly specialized bioinformatic approaches -limited number of experienced laboratories	not recommended outside clinical trials	EuroClonality-NGS working group [20]
ddRQ-PCR	Ig/TCR rearrangement	90–95%	10^−4^ to 10^−5^	-high sensitivity -broad applicability -no need of standard curve	-expensive -limited number of experienced laboratories	not recommended outside clinical trials	-

RQ-PCR, real-time quantitative PCR; RT-gPCR, real-time quantitative reverse transcriptase PCR; ddPCR, digital droplet PCR; NGS, next generation sequencing CHIP, clonal haematopoiesis of indetermined potential; EWALL, European Working Group for Adult Acute Lymphoblastic Leukemia; ALWP EBMT, Acute Leukemia Working Party of the European Society for Blood and Marrow Transplantation.

**Table 2:** A comparison of molecular methods used for measurable residual disease detection in acute myeloid leukemia.

Method	Molecular Target	Applicability	Sensitivity Threshold	Advantages	Limitations	Recommendations for MRD Detection	Standardization
RT-qPCR or RQ-PCR	*NPM1*	30%	10^−4^ to 10^−6^	-High interlaboratory reproducibility -High specificity and sensitivity for leukemic cells -Target stability throughout therapy	-Limited applicability (≤50%)	ELN [1]	EAC [46]
RT-qPCR	*RUNX1-RUNX1T1* *CBF-MYH11 PML-RARA*	1–5% 5% 1–2%					
ddPCR	NPM1 *DNMT3A* *IDH*	30–35%	10^−4^ to 10^−5^	-High sensitivity -The possibility to monitor several mutations simultaneously	-A specific assay needs to be developed for each mutation	Not recommended outside clinical trials	–
NGS	potentially all leukemia-specific genetic aberrations	≥90%	1–5%	-Broad applicability -The ability to study the entire leukemic genome	-Consideration of CHIP at interpretation of results -Requirement of highly specialized bioinformatic approaches -Limited number of experienced laboratories	Not recommended outside clinical trials	–

RQ-PCR, real-time quantitative PCR; RT-gPCR, real-time quantitative reverse transcriptase PCR; ddPCR, digital droplet PCR; NGS, next-generation sequencing CHIP, clonal hematopoiesis of indetermined potential; ELN, EuropeanLeukemia Net; EAC, Europe Against Cancer; EWALL, European Working Group for Adult Acute Lymphoblastic Leukemia; ALWP EBMT, Acute Leukemia Working Party of the European Society for Blood and Marrow Transplantation.
